# Parallel evolution in Ugandan crater lakes: repeated evolution of limnetic body shapes in haplochromine cichlid fish

**DOI:** 10.1186/s12862-015-0287-3

**Published:** 2015-02-04

**Authors:** Gonzalo Machado-Schiaffino, Andreas F Kautt, Henrik Kusche, Axel Meyer

**Affiliations:** Department of Biology, Chair of Zoology and Evolutionary Biology, University of Konstanz, Universitätsstrasse 10, 78457 Konstanz, Germany; International Max Planck Research School for Organismal Biology, University of Konstanz, Universitätsstrasse 10, 78457 Konstanz, Germany

**Keywords:** Parallel evolution, Benthic-limnetic, Speciation

## Abstract

**Background:**

The enormous diversity found in East African cichlid fishes in terms of morphology, coloration, and behavior have made them a model for the study of speciation and adaptive evolution. In particular, haplochromine cichlids, by far the most species-rich lineage of cichlids, are a well-known textbook example for parallel evolution. Southwestern Uganda is an area of high tectonic activity, and is home to numerous crater lakes. Many Ugandan crater lakes were colonized, apparently independently, by a single lineage of haplochromine cichlids. Thereby, this system could be considered a natural experiment in which one can study the interaction between geographical isolation and natural selection promoting phenotypic diversification.

**Results:**

We sampled 13 crater lakes and six potentially-ancestral older lakes and, using both mitochondrial and microsatellite markers, discovered strong genetic and morphological differentiation whereby (a) geographically close lakes tend to be genetically more similar and (b) three different geographic areas seem to have been colonized by three independent waves of colonization from the same source population. Using a geometric morphometric approach, we found that body shape elongation (i.e. a limnetic morphology) evolved repeatedly from the ancestral deeper-bodied benthic morphology in the clear and deep crater lake habitats.

**Conclusions:**

A pattern of strong genetic and morphological differentiation was observed in the Ugandan crater lakes. Our data suggest that body shape changes have repeatedly evolved into a more limnetic-like form in several Ugandan crater lakes after independent waves of colonization from the same source population. The observed morphological changes in crater lake cichlids are likely to result from a common selective regime.

**Electronic supplementary material:**

The online version of this article (doi:10.1186/s12862-015-0287-3) contains supplementary material, which is available to authorized users.

## Background

The spectacular species richness of cichlid fishes and their famous phenotypic diversity in terms of morphology, coloration, and behavior have made them a well-known textbook model system for the study of speciation and adaptive evolution [1-3]. The adaptive radiations of cichlid fishes in East Africa are also renowned for their astonishingly fast rates of speciation [4-7]. The most species-rich endemic cichlid species flocks are made up entirely of species that belong to one particular lineage of cichlids known as the Tribe Haplochromini [2,4,5,8,9]. The adaptive radiation of cichlids in Lake Victoria has attracted particular attention of biologists because its ~500 endemic species probably arose within less than 100,000 years [5,6], which translates to one of the fastest known rates of speciation [10].

Another fascinating aspect of cichlid evolution is the frequent occurrence of evolutionary parallelisms, where species from different lakes have independently evolved a remarkable phenotypic resemblance, converging on several traits, including coloration, body shape, and trophic morphology [1,9,11,12]. Parallel morphological evolution has been considered to be strong evidence for similar regimes of natural selection being at work in driving diversification [13]. By studying repeated parallel evolution, the independent evolution of similar morphologies from a recent common ancestor in isolated and similar environments, we are investigating whether natural selection alone might be sufficient to produce these parallel morphologies, or whether genetic drift, geographic isolation, developmental or genetic bias has influenced the direction of diversification [14-16]. Multiple crater lakes are an ideal system for the study of parallel evolution in body shape in cichlids where similar morphs have repeatedly evolved under comparable ecological niches [17,18].

**Figure 1:**
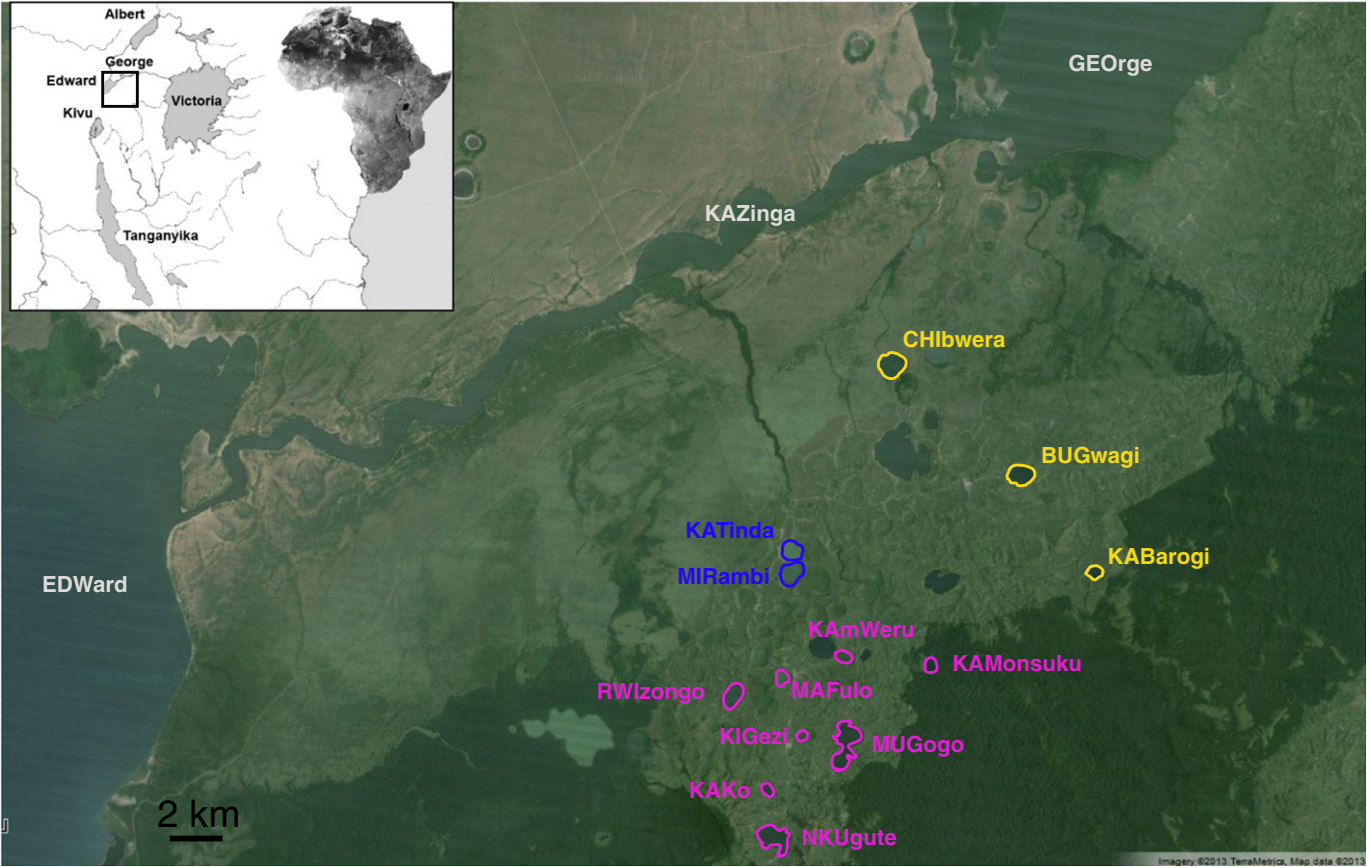
**Map showing the different lakes sampled in southwestern Uganda.** Localities are color coded according to the different identified geographic clusters: gray, yellow, blue and purple for Source, Northern, Central and Southern lakes, respectively. Map source: Google Earth©2014.

The crater lakes in southwestern Uganda (close to the Kazinga Channel that connects Lakes Edward and George, Figure 1) represent one of the few natural experiments in which one can study whether independent parallel diversification took place after independent colonization events. This region of Uganda is biologically almost completely unexplored. Over 50 crater lakes were created in this area by extensive volcanic activity in the East African Great Rift Valley. Geologists date the earliest volcanic activity in this region to approximately 50,000 years ago [19]. Some of these lakes were established through temporal connections with nearby river systems. Until now, only a few of these crater lakes (e.g., Lake Kyamwiga and Lake Nkugute) have been studied [20,21], and each lake was found to contain one genetically and morphologically distinct haplochromine species (*Haplochromis “Lutoto*” in Lake Nkugute and H. *“Nshere”* in Lake Kyamwiga). Analyses using mitochondrial and nuclear markers [20] suggested that these two new species are distinct, but originated from the same founding populations derived from the Kazinga Channel (Figure 1).

Based on these initial findings, it is reasonable to predict that more Ugandan crater lakes might harbor endemic haplochromine cichlid species – making these lakes an interesting natural experiment that permits one to study the interaction between geographical isolation and natural selection promoting phenotypic diversification and speciation. Each crater lake probably provides new and different habitats that are not found in the rivers or great lakes, such as clear and deep open water niches, each of which might exert similar selective regimes.

Here, we report on the first phylogeographical investigation of the haplochromine cichlids of the Ugandan crater lakes combining morphological and population genetic analyses. Using both mitochondrial and microsatellite markers, we inferred the phylogeographic relationships among 16 lakes within the region (Figure 1). Based on this, we also reconstructed the source populations as well as estimated time of colonization for each of the studied crater lakes. We then tested, using geometric morphometrics, whether independently colonized crater lakes, with characteristic larger pelagic zones, promoted the repeated evolution of limnetic-like body shapes from the ancestral deeper-bodied colonizing species.

## Results

### Genetic differentiation

Medium to high levels of genetic polymorphism were detected for most of the microsatellite loci (Table 1). Allelic richness ranged from 2.77 (MAF) to 6.46 (KAZ). Despite the fact that heterologous primers were used, no sign of null alleles was detected. Moreover, no clear signal of balancing or directional selection was detected with LOSITAN software for the panel of 15 microsatellites employed in this study.

**Table 1:** **Genetic diversity based on 15 microsatellites**

**Lake**	**Abbreviation**	**Surface area (km** ^**2**^ **)**	**Coordinates**	**N**	**Allelic richness**	**Gene diversity**	**Fish**
Edward	EDW	2320	0°8'51.80"S 29°52'52.37"E	36	6.24	0.911	0.113
Kazinga	KAZ	-	0°7'30.35"S 30°2'52.96"E	32	6.46	0.932	0.126
George	GEO	250	0°1'48.82"S 30°9'0.91"E	18	5.98	0.895	0.06
Chibwera	CHI	0.87	0°9'2.94"S 30°8'18.18"E	16	5.24	0.824	0.019
Bugwagi	BUG	0.61	0°11'42.72"S 30°11'1.56"E	20	3.65	0.654	0.037
Kabarogi	KAB	0.28	0°13'37.20"S 30°12'43.50"E	21	3.79	0.71	0.016
Katinda	KAT	0.52	0°13'3.42"S 30°6'19.68"E	31	3.83	0.608	0.06
Mirambi	MIR	0.58	0°13'30.30"S 30°6'27.36"E	7	3.06	0.533	0.071
Rwizongo	RWI	0.55	0°16'15.90"S 30°5'20.40"E	18	2.87	0.507	0.096
Mafulo	MAF	0.31	0°16'2.88"S 30°6'14.04"E	19	2.77	0.501	0.168
Kamweru	KAW	0.27	0°15'36.90"S 30°7'19.80"E	21	4.26	0.716	0.012
Kamonsuku	KAM	0.23	0°15'51.36"S 30°9'14.76"E	15	3.07	0.47	0.094
Kigezi	KIG	0.15	0°17'16.26"S 30°6'42.96"E	19	3.77	0.662	0.059
Mugogo	MUG	1.15	0°17'42.72"S 30°7'13.62"E	38	5.02	0.807	0.117
Kako	KAK	0.20	0°18'18.72"S 30°5'53.58"E	5	5.04	0.813	0.016
Nkugute	NKU	1.03	0°19'51.60"S 30°6'12.36"E	11	4.47	0.754	0.121

N = sample size.

**Figure 2:**
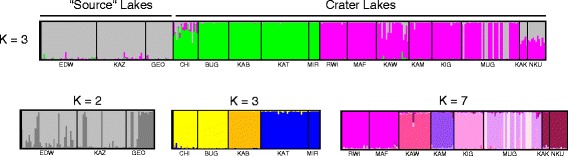
**Bayesian population assignment test based on 15 microsatellite loci with** ***STRUCTURE*** **.** A hierarchical analysis was performed. The most likely number of clusters after DeltaK Evanno’s correction corresponds to K = 3 (source, northern + central and southern lakes represented in gray, green and purple, respectively). Further analyses were performed for each of these clusters separately and the most likely number of clusters is shown.

**Figure 3:**
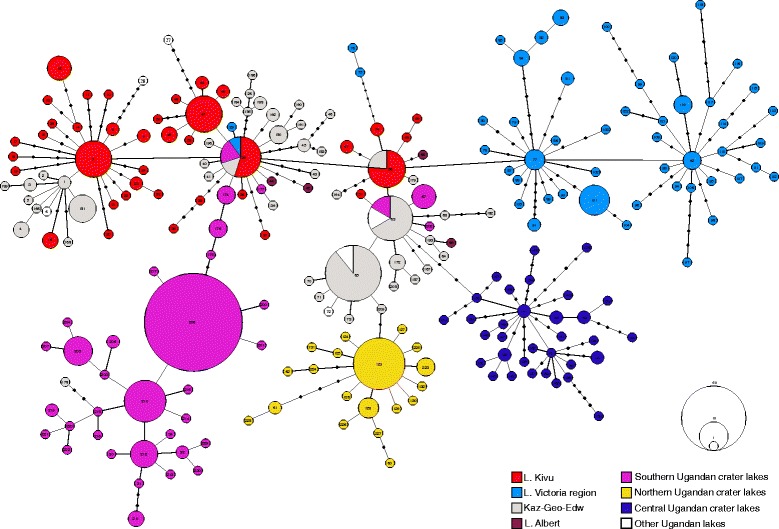
**Median-Joining network showing the relationships among haplotypes, defined by mitochondrial control region sequence variation.** The size of the circles is proportional to the frequency of each haplotype. Haplotypes from Kivu, Victoria, source, northern, central and southern lakes are shown in red, light blue, gray, yellow, blue and purple, respectively.

Strong genetic differentiation was found between crater lakes using both microsatellite and mtDNA markers, in marked contrast to high levels of gene flow between the great Rift Valley Lakes Edwards and George (EDW and GEO) and the Kazinga Channel that connects them (Tables 2 and 3). High levels of genetic differentiation between lakes and regions were also suggested by means of a principal coordinate analysis (Additional file 1) and the clustering pattern determined with STRUCTURE (Figure 2). The most likely number of clusters was determined to be three, following Evanno’s Delta K correction procedure [22]. This suggests that there are three geographically distinct groups: one group composed of both great lakes and the river connecting them (EDW-KAZ-GEO, in gray), one formed by the northern and central crater lakes (in green), and one including the southwestern crater lakes (in purple). Interestingly, further differentiation was detected when each of the previously mentioned clusters was analyzed separately. This is in agreement with high levels of differentiation found even within regions (Table 2). The group affiliations are in concordance with the results found in the haplotype network (see Figure 3).

**Table 2:** **Pairwise genetic differentiation (Fst) based on microsatellite (below) and mtDNA control region loci (above diagonal)**

	**EDW**	**KAZ**	**GEO**	**CHI**	**BUG**	**KAB**	**KAT**	**MIR**	**RWI**	**MAF**	**KAW**	**KAM**	**KIG**	**MUG**	**KAK**	**NKU**
EDW	-	0.176*	0.150*	0.405**	0.486**	0.535**	0.375**	0.471**	0.362**	0.666**	0.486**	0.699**	0.651**	0.382**	0.490**	0.615**
KAZ	0.021^ns^	-	0.168^ns^	0.217*	0.257*	0.275**	0.287**	0.220^ns^	0.243*	0.381**	0.307**	0.396**	0.375**	0.311**	0.217^ns^	0.328*
GEO	0.101**	0.039^ns^	-	0.512**	0.611**	0.725**	0.378**	0.619**	0.607**	0.881**	0.658**	0.866**	0.863**	0.497**	0.711**	0.815**
CHI	0.378**	0.361**	0.370**	-	0.106**	0.180**	0.441**	0.647**	0.653**	0.868**	0.687**	0.853**	0.852**	0.592**	0.711**	0.803**
BUG	0.523**	0.488**	0.481**	0.147**	-	0.008 ns	0.468**	0.749**	0.749**	0.928**	0.761**	0.909**	0.914**	0.650**	0.823**	0.879**
KAB	0.488**	0.482**	0.502**	0.276**	0.374**	-	0.504**	0.864**	0.817**	0.982**	0.813**	0.958**	0.968**	0.688**	0.927**	0.943**
KAT	0.529**	0.510**	0.523**	0.239**	0.302**	0.220**	-	0.079^ns^	0.472**	0.609**	0.470**	0.650**	0.595**	0.396**	0.361**	0.560**
MIR	0.589**	0.578**	0.617**	0.279**	0.547**	0.324**	0.146^ns^	-	0.669**	0.914**	0.631**	0.872**	0.885**	0.505**	0.648*	0.807**
RWI	0.423**	0.430**	0.511**	0.436**	0.666**	0.589**	0.658**	0.754**	-	0.504**	0.141^ns^	0.749**	0.468**	0.254**	0.581**	0.593**
MAF	0.468**	0.491**	0.559**	0.529**	0.728**	0.687**	0.723**	0.782**	0.147**	-	0.091^ns^	0.928**	0.001^ns^	0.346**	0.908**	0.833**
KAW	0.324**	0.318**	0.328**	0.334**	0.514**	0.545**	0.588**	0.669**	0.367**	0.354**	-	0.649**	0.067^ns^	0.159*	0.406*	0.411**
KAM	0.556**	0.560**	0.579**	0.577**	0.747**	0.736**	0.737**	0.894**	0.660**	0.559**	0.316**	-	0.894**	0.548**	0.839**	0.834**
KIG	0.459**	0.462**	0.475**	0.322**	0.546**	0.565**	0.584**	0.741**	0.525**	0.525**	0.130**	0.493**	-	0.318**	0.836**	0.763**
MUG	0.231**	0.232**	0.250**	0.283**	0.439**	0.523**	0.535**	0.571**	0.339**	0.321**	0.043^ns^	0.248**	0.124*	-	0.247^ns^	0.252^ns^
KAK	0.353**	0.347**	0.297**	0.425**	0.645**	0.627**	0.649**	0.843**	0.577**	0.499**	0.084^ns^	0.427**	0.325**	0.070^ns^	-	0.704**
NKU	0.450**	0.480**	0.481**	0.553**	0.728**	0.707**	0.718**	0.845**	0.611**	0.467**	0.284**	0.323**	0.463**	0.178*	0.205**	-

NS, not significant; *P < 0.05 and **P < 0.01 after sequential Bonferroni correction [70].

**Table 3:** **Effective migrants estimated with MIGRATE (five independent runs) based on microsatellite data**

**Source and receiving pop**	**N**_**e**_ **m**	**95% CI**
KAZ → EDW	7.82	6.97 - 8.92
EDW → KAZ	3.63	3.17 – 4.07
KAZ → GEO	2.38	2.08 – 2.74
GEO → KAZ	3.28	2.84 – 3.78
KAZ → CHI	5.90	5.22 – 6.68
CHI → KAZ	5.32	4.62 – 6.27
KAZ → BUG	6.86	5.82 – 8.10
BUG → KAZ	0.15	0.12 – 0.18
KAZ → KAB	10.09	8.74 – 11.64
KAB → KAZ	0.53	0.46 – 0.61
KAZ → KAT	9.47	8.33 – 11.29
KAT → KAZ	1.75	1.50 – 1.99
KAZ → RWI	7.49	6.48 – 9.19
RWI → KAZ	0.69	0.59 – 0.80
KAZ → MAF	1.99	1.68 – 2.38
MAF → KAZ	0.22	0.18 – 0.26
KAZ → KAW	1.55	1.28 – 1.91
KAW → KAZ	0.42	0.36 – 0.53
KAZ → KAM	3.08	2.53 – 3.59
KAM → KAZ	1.98	1.68 – 2.39
KAZ → KIG	1.26	1.08 – 1.48
MUG → KAZ	0.52	0.44 – 0.62
KAZ → MUG	14.19	12.61 – 16.03
MUG → KAZ	1.26	1.12 – 1.42

Central haplotypes in the haplotype network were almost exclusively from great lakes Edward and George (EDW-KAZ-GEO), supporting the hypothesis that these are the ancestral populations (Figure 3). Moreover, three different mitochondrial lineages (northern, central and southern haplotypes are shown in yellow, blue and purple, respectively) were found corresponding to the geographically defined groups of crater lakes. Most of the haplotypes found in the crater lakes were only a few mutations apart from the ancestral ones, providing evidence that these populations have diverged rather recently.

### Colonization time

Most of the crater lake populations showed a distinctive pattern of population expansion (Fu’s tests were significantly negative), probably following colonization (Table 4). However, this pattern was not so clear in the great lakes Edward and George, where the population sizes seem to be more stable.

**Table 4:** **Demographic changes inferred using mitochondrial control region**

	**EDW**	**KAZ**	**GEO**	**CHI**	**BUG**	**KAB**	**KAT**	**MIR**	**RWI**	**KAW**	**KAM**	**KIG**	**MUG**	**KAK**	**NKU**
*Mean Number of differences*	4.221	17.957	1.895	2.608	0.830	0.200	4.953	1.714	2.275	2.914	0.819	0.316	3.572	1.400	0.873
*Tau*	4.998	40.883	0.001	0.914	0.930	3.000	4.977	2.000	3.580	0.001	1.123	3.000	8.621	1.844	0.914
*T (MYA)*	0.093	0.758	0.000	0.017	0.017	0.056	0.092	0.037	0.066	0.000	0.021	0.056	0.160	0.034	0.017
*Theta0*	0.001	0.001	0.001	0.001	0.001	0.001	0.001	0.001	0.002	0.001	0.004	0.001	0.001	0.001	0.001
*Theta1*	35.508	7.765	103.525	99999	99999	0.255	99999	99999	4.985	99999	2.705	0.235	3.477	99999	99999
*SSD*	0.015^ns^	0.040^ns^	0.489^**^	0.133^*^	0.004^ns^	0.002^ns^	0.002^ns^	0.055^ns^	0.024^ns^	0.309^**^	0.005^ns^	0.008^ns^	0.094^ns^	0.068^ns^	0.001^ns^
*Raggedness Index*	0.031^ns^	0.018^ns^	0.053^ns^	0.062^ns^	0.091^ns^	0.413^ns^	0.021^ns^	0.234^ns^	0.069^ns^	0.165^ns^	0.064^ns^	0.493^ns^	0.206^ns^	0.350^ns^	0.063^ns^
*Fu’s Fs*	−7.842^**^	0.530^ns^	−1.599^ns^	−3.075^*^	−6.083^**^	−1.863^*^	−25.42^**^	−3.709^**^	−0.108^ns^	0.0311^ns^	−2.041^*^	−1.085^*^	1.203^ns^	−1.648^*^	−1.026^ns^

Fs = Fu’s test of neutrality. SSD tests the validity of a stepwise expansion model based on the sum of squares deviations between the observed and expected mismatch, non-significant mismatch values suggest population expansion. Raggedness Index is calculated similarly, non-significant raggedness values suggest population expansion. Time since lineage expansion (t) is calculated from Tau = 2μt, where μ = 3.25%/MYR for 830 bp. Significance level: NS, non-significant; *P < 0.05 and **P < 0.001.

Clear asymmetric gene flow was detected for all the crater lakes, with, as expected, a much higher effective migration from the great lakes (EDW-KAZ-GEO) to the crater lakes than in the other direction (Table 3). This pattern reinforces the idea that the great lakes George and Edward were the older, larger and ancestral populations for the haplochromine cichlids that subsequently colonized the crater lakes of southwest Uganda.

Estimation of divergence times, together with the fact that three different mitochondrial lineages were found (see Figure 3), suggests that there were at least three independent waves of colonization from the source population (Table 5). The oldest colonization event took place around 89,000 years ago to the crater lakes located geographically closer to the source lakes, those in the center (CEN) of the study area (KAT and MIR). Fittingly, these lakes also contain the largest haplotype diversity among the haplochromine cichlids sampled (Figure 3). The older age of these crater lake haplochromine populations is also supported by the presence of many exclusive alleles and the fact that their private haplotypes are separated by several mutations in the haplotype network (Figure 3). The crater lakes located in the northern region of the study area (CHI, BUG, KAB) were colonized around 71,000 years ago, whereas it seems that the geographically more distant lakes (southern part of the study area) were colonized more recently, around 50,000 years ago. This pattern is also supported by the fact that southern crater lakes share some central haplotypes with the great lakes, suggesting a more recent divergence or several colonization events. Based on the phylogenetic analyses of the microsatellite data, all southern crater lakes grouped together in a NJ tree and were clearly separated from the central and northern lakes (Additional file 2).

**Table 5:** **Divergence times and effective migration rates estimated using a coalescent approach from the mitochondrial control region data (MDIV software)**

	**Location**	**KAZ vs.**	**T (μ = 0.057)**	**M**	**T (μ = 0.0325)**
Great lake	*Source*	EDW	7.0 (0–14.2)	6.312 (1.080)	12.3 (0–25)
Great lake	*Source*	GEO	4.5 (0–10.2)	7.671 (0.216)	7.9 (0–17.7)
Crater lake	*North*	BUG	49.0 (42.3 - 55.7)	0.009 (0.006)	85.9 (74.1 - 97.7)
Crater lake	*North*	CHI	82.6 (79.5 - 85.7)	0.008 (0.006)	144.9 (139.2 - 150.6)
Crater lake	*North*	KAB	46.6 (41.5 - 51.7)	0.025 (0.034)	81.8 (73–90.6)
Crater lake	*Central*	KAT	94.3 (90.3 - 98.2)	0.003 (0.001)	165.4 (158.3 - 172.5)
Crater lake	*South*	KAM	56.8 (53.3 - 60.3)	0.009 (0.006)	99.6 (93.5 - 105.7)
Crater lake	*South*	KAW	44.5 (37.2 - 51.8)	0.989 (0.076)	78.1 (65.6 - 90.6)
Crater lake	*South*	MAF	60.5 (51.4 - 69.1)	0.009 (0.007)	106.2 (90.9 - 121.5)
Crater lake	*South*	RWI	43.1 (34.3 - 51.9)	0.251 (0.018)	75.6 (60.3 - 90.9)
Crater lake	*South*	NKU	47.6 (39–56.2)	0.004 (0.001)	83.4 (68.3 - 98.5)
Group 1	*North*	CHI + BUG + KAB	71.5 (62.6 - 80.4)	0.0006 (0.0004)	125.4 (109.8 - 141)
Group 2	*Central*	KAT + MIR	89.0 (75.8 - 102.2)	0.002 (0.001)	156.1 (133–179.2)
Group 3	*South*	Southern craters	54.4 (50.5 - 58.3)	0.687 (0.049)	95.4 (88.6 - 102.2)

Estimates are based on four independent runs. T in thousands of years (95% confidence interval), M = 2 Ne m. Group 1, 2 and 3 correspond to the different mitochondrial lineages suggested from Figure 3.

### Morphological differentiation

**Figure 4:**
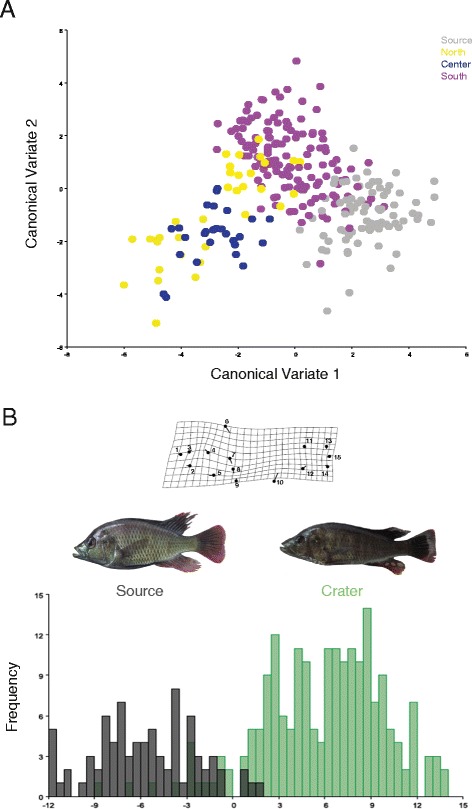
**Body shape differentiation between Haplochromine cichlids from source and crater lakes. (A)** Canonical variate analysis. **(B)** Cross validation analysis between source and crater lakes. Shape changes along CV 1 and 2 are indicated by thin plate splines (scale factor = 2). The line terminus refers to the shape change along a particular axis, compared with the average shape (black dot).

No morphological differentiation was detected among fish collected from the great lakes (EDW-KAZ-GEO). However, a clear pattern of morphological differentiation was found between the haplochromine cichlids from the source and crater lake populations. No overlap was detected in the measured morphospace between the great lakes and the crater lakes except in a few individuals from the southern lakes (Figure 4A).

Only one morphometric cluster was found for each crater lake, suggesting no intralacustrine differentiation. Nevertheless, we found that haplochromine cichlids from the source and the young crater lakes have significantly different body shapes (Hotelling’s T^2^ test, P < 0.0001). Crater lake cichlids have more shallow body shapes (Landmarks 6, 9 and 10) relative to the great lake source cichlids (see the thin plate spline representation in Figure 4 B and Additional file 3). Interestingly, this pattern of morphological differentiation between source and crater lakes was consistent for most of the pairwise comparisons (Additional file 3) providing evidence for repeatedly evolved limnetic body shapes.

A positive correlation (r = 0.448, P = 0.052) between genetic (Fst) and morphometric (Procrustes) distance was found, suggesting that younger crater lakes (e.g. southern lakes) are more similar to the source populations.

## Discussion

We found clear evidence for strong genetic and morphological differentiation of haplochromine cichlid fishes from the crater lakes and the source lakes (Edward and George) in Uganda. Based on mitochondrial markers, at least three different waves of colonization were suggested to have occurred, coinciding with increased geological activity around 50,000 years ago [19]. In addition to genetic differences, morphological differentiation was associated with the colonization of the Ugandan crater lakes. Crater lake fish are more slender than those from the shallow source lakes. Thus, the repeated evolution of elongated body shapes is likely to be an adaptation to living in the open, clear and deep waters of crater lakes compared to the murky and shallow waters of their ancestral lakes and riverine habitats.

### Genetic differentiation

As expected, higher levels of genetic diversity were found in the great lakes than in the much younger crater lakes. The degree of genetic differentiation between crater lakes, even among those from the same region, supports a scenario in which each of the crater lakes constitutes a geographically isolated population. Moreover, no intralacustrine differentiation was detected, and each crater lake (except for Mugogo) consists of only one genetic cluster based on the genetic marker set employed.

Although based on only a relatively small number of individuals (N = 38), clear genetic differentiation was found between riverine and crater lake haplochromine cichlids in Lake Mugogo (see STRUCTURE plot for Lake Mugogo, MUG in Figure 2), suggesting no gene flow between riverine and crater lake populations. Consequently, our results indicate that these populations diverged mainly in allopatry, which is generally considered to be the most common and plausible mode of speciation [23-25].

Clear genetic and morphological differences exist between several lakes, suggesting that some of these crater lakes likely harbor undescribed and endemic haplochromine species. These findings should be corroborated by further taxonomic investigations.

### Colonization/parallelism

The observed patterns of genetic differentiation and asymmetric gene flow found with both kinds of genetic markers support at least three independent colonization events from the great lakes into sets of geographically-clustered crater lakes. This finding is in line with evidence from previous studies that suggest that two crater lakes within this region were colonized by the Edward-Kazinga-George system [20]. Moreover, several central haplotypes were shared with those found in the older Lake Kivu, which was suggested to be ancestral to Lake Victoria [6]. Interestingly, the central haplotype 56 that connects Lake Kivu (LK) with Lake Victoria was suggested to be exclusive to lake Kivu in previous studies, supporting the crucial role of Lake Kivu haplochromines in the evolution of the haplochromines of eastern Africa [6]. However, in the present study we did find that this haplotype is also present in the Edward-Kazinga-George system, highlighting the relevance of this region during the stepwise colonization by haplochromine cichlids of the Lake Victoria region from the Lake Kivu region.

Strikingly, every independent colonization event of Ugandan crater lake cichlids was associated with a morphological change in the same direction - that is, the evolution of a more slender (limnetic-like) body shape. An elongated, more streamlined body is usually associated with the exploitation of open water habitats [26-28]. Moreover, the repeated pattern of phenotypic divergence in concert with the use of certain habitats has been taken as evidence for the important role of natural selection in the generation of diversity [29,30]. Alternatively, only a limited set of phenotypes might be obtained in evolution, and the entire morphospace is not available for all lineages [14,31,32]. Indeed, an eco-morphological differentiation along the limnetic-benthic axis is taxonomically widespread and has long been reported as the most common pattern of divergence in freshwater fishes [28,33-36]. Also, divergence along a benthic to limnetic axis is common in cichlids [17,37-40] and we are beginning to identify the genomic basis for such ecologically divergent body shapes [41]. In our case, even though phenotypic plasticity cannot be completely ruled out, it does not explain much of the variance in shape found when wild larvae fish are reared in the lab (see Additional file 4). Clear morphological differentiation between lab-reared crater lake fish and fish from the source lakes was found (Hotelling’s T^2^ test, P < 0.001, Additional file 4). Thus, crater lake fish reared in lab conditions maintain shallower body shapes than those of wild fish captured in the great lakes. Altogether, this would suggest a genetic component of the elongated body shape characteristic of Ugandan crater lake cichlids.

A scenario in which the riverine haplochromine had first differentiated into more elongated shape and later colonized the crater lakes cannot be completely discarded, however, it is rather unlikely due to the fact that all the haplochromine cichlids found in the ancestral lakes (including the river Kazinga) have deeper body shapes, and that each of the three mitochondrial lineages (waves of colonization) originated from the central haplotypes (Figure 3, in gray). Moreover, riverine species inhabiting the proximity of the crater lakes were genetically different from the crater lake populations (see the STRUCTURE plot for Lake Mugogo, MUG in Figure 2). Unfortunately, the low number of riverine specimens included in this study precluded the proper geometric morphometric comparison between crater lake and riverine individuals.

### No intralacustrine diversification

Although clear genetic and morphological differentiation was found between each crater lake and the great lakes, no signal of intra-lacustrine diversification was detected. Different factors, such as temporal and spatial variation, ecological opportunity, and lineage-specific features have been proposed to affect the propensity for intralacustrine radiation [1,42,43].

Obviously, linage-specific characteristics do not seem to cause the absence of divergence within Ugandan crater lakes due to the fact that members of the same tribe, the haplochromine cichlids, have undergone some of the greatest radiations in other African lakes [2,4,5].

A positive correlation between the size of the lake and its species richness is expected [17,42]. In general, one would expect that the area of a lake is positively correlated with higher environmental heterogeneity. Hence, niche diversity would tend to increase with size as well as the opportunity for isolation by distance (but see Wagner *et al.* [44] 2012). Even though Ugandan crater lakes are generally very small (<1 km^2^_,_ see Table 1), intralacustrine diversification has been found even in smaller crater lakes such as those in Cameroon [45] and Nicaragua [46,47]. The estimates of divergence times are similar to those previously calculated for two other Ugandan crater lakes [20]. It might be expected that because colonization occurred so recently (around 50,000 years ago), there has not been enough time to complete speciation within each lake. However, intralacustrine divergence in cichlid fishes has been detected in much younger lakes, such as Neotropical and African crater lakes, where ecological speciation has been suggested [48,49]. Possible reasons for the perceived lack of intralacustrine diversification in the Ugandan crater lakes might be that deep, clear open-water niches, like those found in the Nicaraguan crater lakes [47], might be much smaller or missing in the very small and relatively shallow crater lakes of Uganda.

## Conclusions

A pattern of strong genetic and morphological differentiation was observed in the Ugandan crater lakes, suggesting that this system might still harbor several undescribed endemic species. The patterns of colonization events suggest that lakes that are geographically close tend to be genetically more similar, and that crater lakes in three different geographic areas have been colonized by three independent waves of colonization. Our data suggest that body shape changes have repeatedly evolved into a more limnetic-like form in several of these natural replicates. The observed morphological changes in Ugandan crater lake cichlids are likely to result from a common selective regime.

## Methods

### Sampling

A total of 337 haplochromine cichlids were collected (sample collection permit FISH201011/AU1) from 13 different lakes in southwestern Uganda, from Lakes Edward and George, and the Kazinga Channel (Figure 1 and Table 1) in November 2011. Fish from both the shore and the middle of the lakes were sampled whenever possible in order to have representative samples from each lake. In addition, riverine haplochromine fish were also collected from a river close to the crater lake Mugogo. Fish were caught using seine nets and hand nets with the assistance of local fishermen. Each fish was euthanized with an overdose of MS-222, labeled and photographed in the field. A small tissue sample was preserved in pure ethanol and stored at 4°C until DNA extraction. These specimens were combined with previously-collected samples from Lakes Victoria, Albert and Kivu [6] stored in Axel Meyer’s collection at the University of Konstanz.

### DNA extraction and amplification

Total DNA was extracted from 1 mm^3^ tissue using a proteinase K digestion followed by sodium chloride extraction and ethanol precipitation [50]. All samples were genotyped for 15 microsatellite loci: Abur25, Abur30, Abur51, Abur82, Abur94, Abur162, Abur165 [51]; OSU19, OSU20 [52]; TmoM5, TmoM7, TmoM11, TmoM27 [53]; UNH001, UNH002 [54]. All loci were PCR amplified with fluorescent reverse primers (HEX, FAM and NED dyes) and fragment length was analyzed with an internal size marker, Genescan-500 ROX (Applied Biosystems), on an ABI 3130 Automated Sequencer (Applied Biosystems) and scored with GeneMapper v4.0 (Applied Biosystems) software.

The mitochondrial control region (CR) (838 bp) was PCR amplified using published primers and reaction conditions (L-Pro-F [55]; 12S5R, 5′-GGC GGA TAC TTG CAT GT-3’) on a GeneAmp PCR System 9700 Thermocycler (Applied Biosystems). The PCR products were purified using the QIAquick PCR Purification kit (QIAGEN), and sequenced in both directions with the BigDye Terminator Cycle Sequencing Ready Reaction kit (Applied Biosystems). Sequencing products were analyzed on an ABI 3130 Automated Sequencer (Applied Biosystems). Mitochondrial DNA sequences were aligned using the software SEQUENCHER v. 4.2 (Gene Code Corporation) and verified by eye.

### Mitochondrial data analysis

Mitochondrial CR sequences were edited using the BioEdit Sequence Alignment Editor software [56] and aligned with the ClustalW application included in BioEdit. The different haplotypes were obtained with the program DNASP [57] and submitted to Genebank (Accession number KP406813 - KP406919). MODELTEST v3.7 [58] was employed to determine the model of sequence evolution that best fit the datasets and to calculate the proportion of invariable sites and the value of the gamma distribution shape parameter.

Mitochondrial variation was analyzed with the program ARLEQUIN version 3.01 [59], weighting 1:2 transitions and transversions, respectively. Within-species variation was estimated by nucleotide diversity (π) and haplotype diversity (h) [60]. Haplotype networks were inferred with HAPSTAR v. 0.7 [61] based on connection lengths calculated in ARLEQUIN [62].

Deviations from equilibrium were tested with Fu’s Fs [63] neutrality tests based on infinite-site model without recombination. Negative values of Fu’s F are expected under a model of sudden population expansion. Mismatch distributions [64] were also calculated to investigate demographic changes. Time since lineage expansion (t) was calculated from tau = 2 μt, where t is the expansion time and μ is the mutation rate per million years per nucleotide.

Divergence times and migration rates among source and crater lake populations were estimated by comparing mitochondrial sequences with the program MDIV [65]. Initial runs were tested under a finite sites (HKY) model of evolution and default priors (M = 10, T = 5) to approximate the posterior distribution of scaled migration rate (M) and time since divergence (T), while allowing MDIV to estimate θ. We ran the MCMC for 5 million generations with 600,000 generations discarded as burn-in. Convergence was determined by evaluating the consistency of model values for each of the three parameters across four runs, which were then averaged to calculate mean θ, M and T values ± standard deviation. Time of divergence was calculated as tdiv = Tθ/2Lμ [65] where T (or TMRCA) and θ were estimated by the height of the posterior distribution, L is the sequence length analyzed, and μ is the mutation rate. Divergence times were estimated based on two different substitution rates [66] (see Table 5).

### Microsatellite data analysis

Scoring errors, large allele dropout and null alleles were checked in MICROCHECKER [67]. The LOSITAN software [68] was used in order to test for neutrality. Microsatellite variation (allelic richness per locus, observed and expected heterozygosity) was calculated with the program GENETIX 4.05 [69].

The program ARLEQUIN [59] was employed for estimates of F_ST_ values and their statistical significance between samples pairs, i.e. the significance of population differentiation, with the following settings: 1000 permutations for significance, 10,000 steps in Markov chain. Levels of significance for multiple tests were determined using sequential Bonferroni adjustments for simultaneous tests [70] whenever relevant.

The software STRUCTURE v2.3 [71] was used to assess the number of genetic clusters (K) using a Bayesian approach. A burn-in period of 50,000 steps followed by 500,000 Markov chain Monte Carlo (MCMC) iterations were enough to ensure convergence. Five independent runs were performed using an admixture (each individual draws some fraction of its genome from each of the K populations) and correlated allele frequencies model. The STRUCTURE software provides an estimation of the membership fraction in each of the inferred clusters. A hierarchical structure analysis was performed until no more resolution was observed. Evanno’s correction [22] was implemented and visualized using STRUCTURE HARVESTER [72] software. Thus, Delta K values were used to infer the most likely number of genetic clusters (K) at each hierarchical level. Genetic clusters were further validated using a principal coordinate analysis (PCoA) based on genetic distance in GenAlEX [73].

The program MIGRATE-N version 3.5.1 [74] was then used to estimate dispersal rates and long-term effective population size (N_e_) from the microsatellite data, using a maximum-likelihood (ML) coalescent approach and averaging over five runs. The program estimates Ө, which is the product of the effective population size and mutation rate: 4N_e_μ, where μ is the mutation rate per generation. The effective number of migrants per generation, N_e,_ is estimated as well as migration rate, m. As program settings, we employed a stepwise-mutation model (Brownian motion approximation) and used the default settings for other parameters. For each run, starting estimates for Ө were based on F_ST_ values, with a burn-in of 15,000 trees, 14 short chains with a total of 100,000 genealogies sampled, and three long chains with 1,000,000 genealogies sampled, for each locus. Adaptive chain heating, with four different temperatures, was used to achieve an efficient exploration of the data.

A Neighbor-Joining tree was constructed based on a distance matrix calculated from the frequency data for the 15 microsatellite loci employing the computer package PHYLIP [75]. Statistical support of nodes was estimated with 1000 bootstrap replicates.

### Morphological analysis: body shape

We examined body shape differentiation among source and crater lake fishes using geometric morphometrics. Fifteen homologous body landmarks were digitized in TPSDIG2.17 [76] from standardized pictures of 370 individuals (see Figure 4 for landmark description).

Shape analyses were performed in MorphoJ1.03d [77]. Landmarks were first aligned using a full Procrustes superimposition, which involves scaling all shapes to unit centroid size, translation to a common position, and rotation to minimize the Procrustes distance between landmark configurations [78,79]. Allometry is common in fish and thus morphology and total body size are typically related [79]. Therefore, a multivariate regression of body shape (Procrustes coordinates) on size (centroid size) was used to correct for allometric effects. Regression residuals were then used for all downstream geometric morphometric analyses.

Individual variation in body shape across and within lakes was visualized using Canonical Variate Analysis (CVA) and Discriminant Function Analysis (DFA) on the regression residuals (Figure 4). Shape differences between groups were visualized using thin plate splines [78]. Body shape differentiation of source and crater lakes was assessed with Hotelling’s T^2^ test as implemented in MorphoJ.

### Availability of supporting data

The data sets supporting the results of this article are available in LabArchives, https://mynotebook.labarchives.com/share/Uganda_BMC-Evol-Biol/MjAuOHw2NzM5Mi8xNi9UcmVlTm9kZS83MTA5NDY5OTl8NTIuOA==.

## Additional files

Additional file 1:
**Principal coordinates analysis based on genotypic data from 15 microsatellites loci showing the genetic differentiation among the four considered groups.** Analyses were performed using the covariance matrix with data standardization of genetic distance using GenAlEx. Additional file 2:
**Neighbor-joining tree estimated from allele frequency data of 15 microsatellite loci for different lakes.** Lake Kivu (KIV) was employed as an outgroup. Additional file 3:
**Pairwise body shape differentiation among source and crater lakes.** Shape changes along CV 1 or 2 are indicated by thin plate splines (scale factor = 2). The line terminus refers to the shape change along a particular axis, compared with the average shape (black dot). Some pairwise comparisons are not shown due to low sample sizes. Additional file 4:
**Body shape differentiation among lab-reared (n = 25), source (n = 86) and crater lake (n = 241) Haplochromine cichlids.** A) Canonical variate analysis. B) Cross validation analysis between source and lab-reared fish.
