# Supplementary figures and images for: Parallel evolution in Ugandan crater lakes: repeated evolution of limnetic body shapes in haplochromine cichlid fish
Source: BMC Evol Biol. 2015 Feb 4;15(1):9. doi: 10.1186/s12862-015-0287-3 (PMC4322459; doi:10.1186/s12862-015-0287-3)

Principal Coordinates (PCoA)

Coord. 2

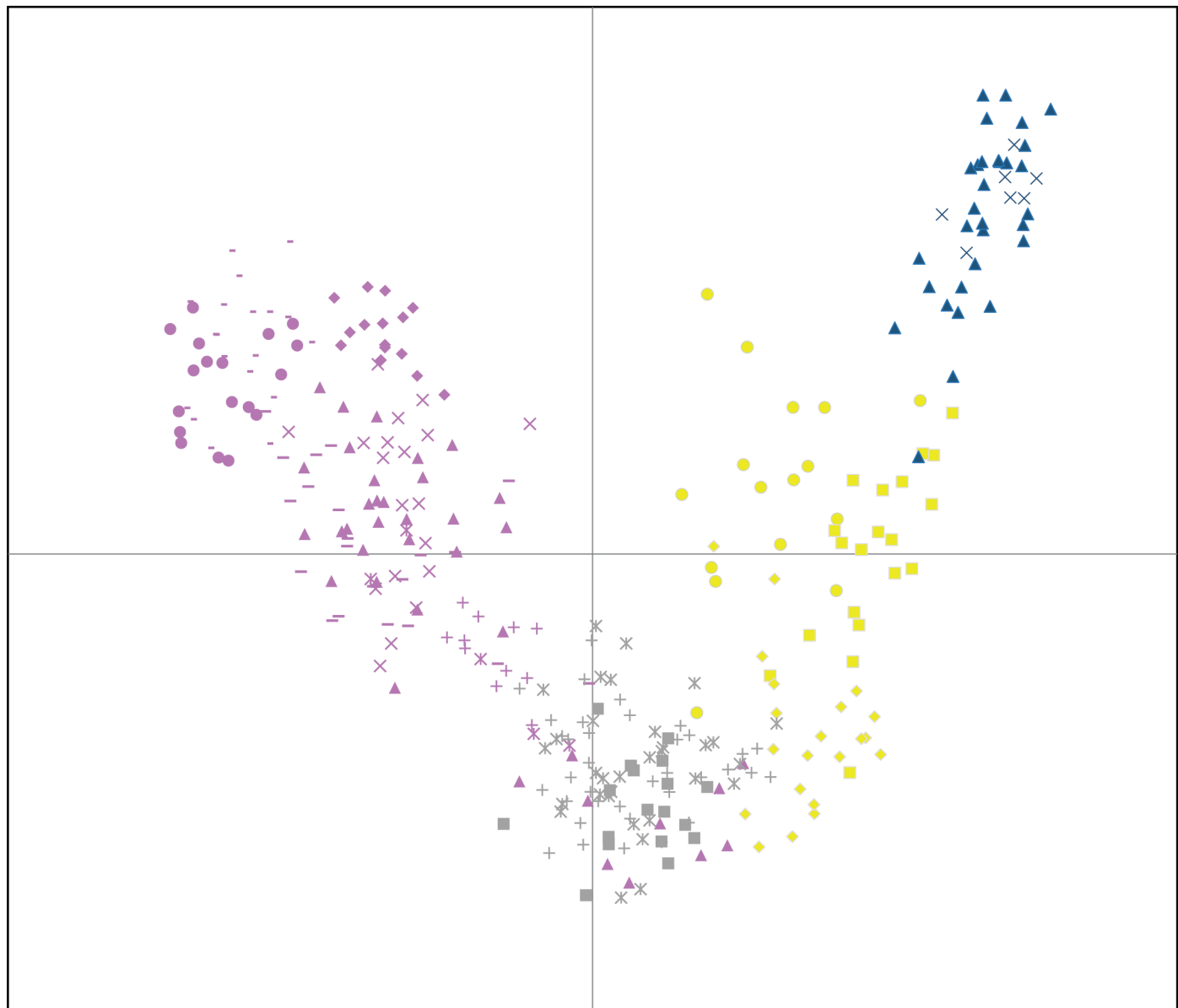

Coord. 1

Supplement: Additional file 1: — Principal coordinates analysis based on genotypic data from 15 microsatellites loci showing the genetic differentiation among the four considered groups. Analyses were performed using the covariance matrix with data standardization of genetic distance using GenAlEx. [file 12862_2015_287_MOESM1_ESM.pdf]

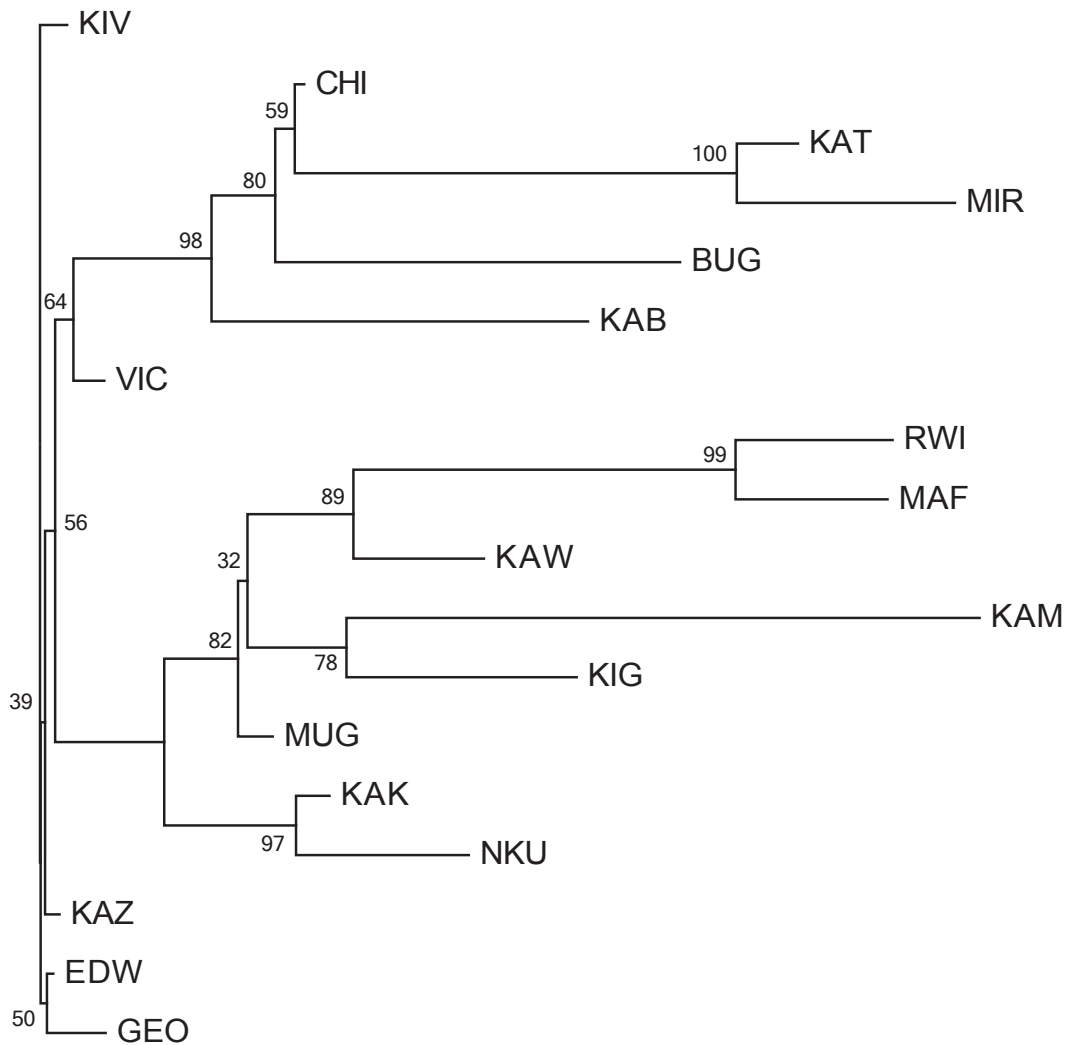

Supplement: Additional file 2: — Neighbor-joining tree estimated from allele frequency data of 15 microsatellite loci for different lakes. Lake Kivu (KIV) was employed as an outgroup. [file 12862_2015_287_MOESM2_ESM.pdf]

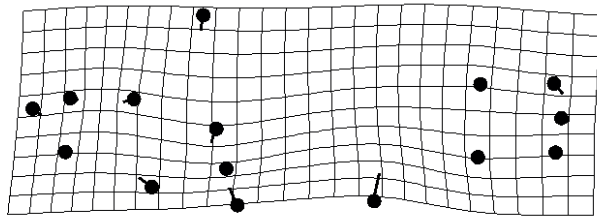

anc -- kab

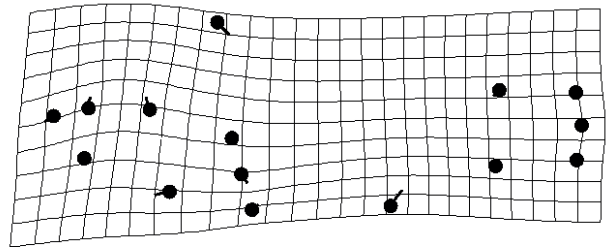

anc -- chi

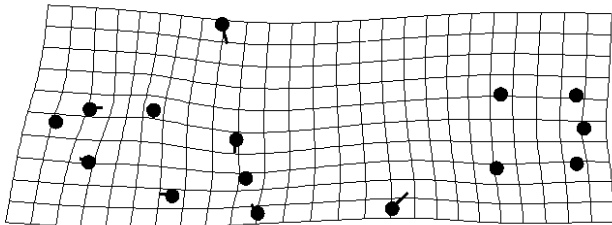

anc -- kat

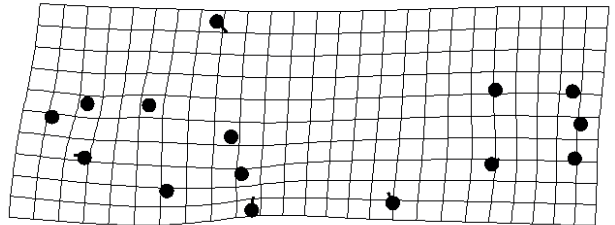

anc -- mug

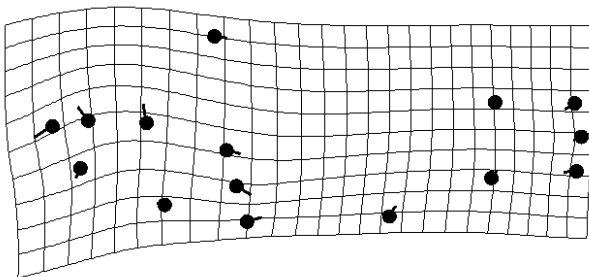

anc -- nku

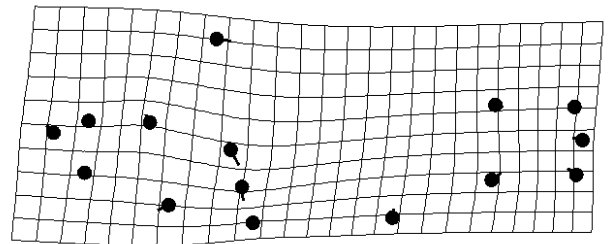

anc -- kig

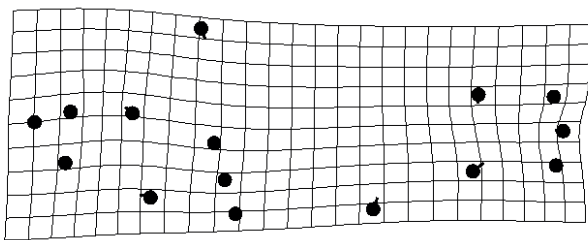

anc -- kaw

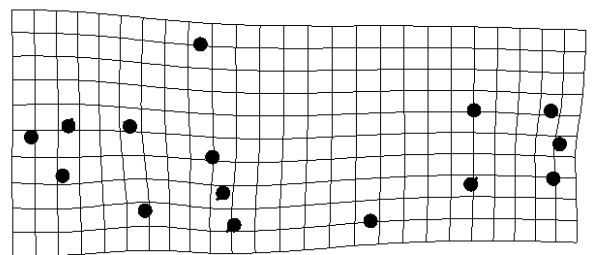

anc -- maf

Supplement: Additional file 3: — Pairwise body shape differentiation among source and crater lakes. Shape changes along CV 1 or 2 are indicated by thin plate splines (scale factor = 2). The line terminus refers to the shape change along a particular axis, compared with the average shape (black dot). Some pairwise comparisons are not shown due to low sample sizes. [file 12862_2015_287_MOESM3_ESM.pdf]

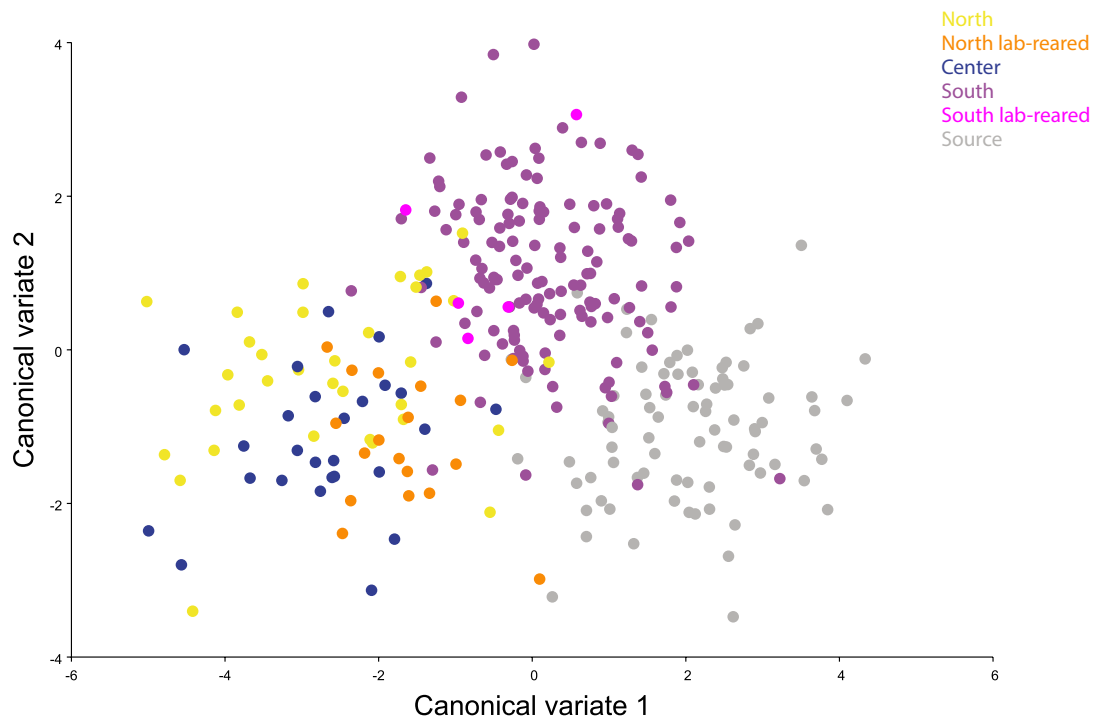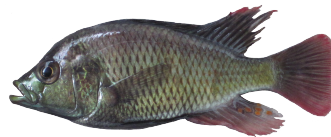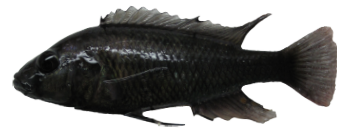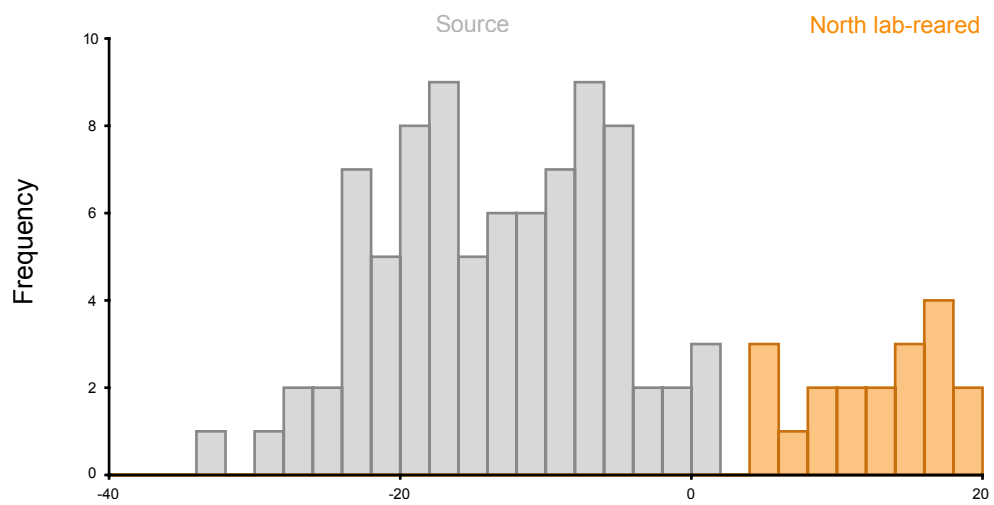

Supplement: Additional file 4: — Body shape differentiation among lab-reared (n = 25), source (n = 86) and crater lake (n = 241) Haplochromine cichlids. A) Canonical variate analysis. B) Cross validation analysis between source and lab-reared fish. [file 12862_2015_287_MOESM4_ESM.pdf]
